# Ultrasensitive Direct Chemical Analysis of Human Hair Using Proton Transfer Reaction Time-of-Flight Mass Spectrometry (PTR-TOF-MS) for Nontargeted Exposure Profiling

**DOI:** 10.1021/acs.chemrestox.5c00002

**Published:** 2025-09-08

**Authors:** Anna C. Neville, David A. Jarma, Daniel C. Blomdahl, Chou-Hsien Lin, Kerry A. Kinney, Pawel K. Misztal

**Affiliations:** Maseeh Department of Civil, Architectural and Environmental Engineering, 12330 University of Texas at Austin, Austin, Texas 78712, United States

## Abstract

Exposure to air pollution plays a significant role in human health. Current methods of measuring human exposure are often limited to outdoor measurements, are time intensive, or are unable to accurately measure certain classes of compounds. This study proposes human hair as a promising indicator of pollution exposure. We present a novel method of hair analysis involving thermal extraction and detection of semivolatile organic compounds using a Vocus 2R proton transfer reaction time-of-flight mass spectrometer (Vocus PTR-TOF-MS). The hair samples were subjected to a temperature ramp spanning three different temperatures: 60 °C, 90 °C, and 120 °C. A hierarchical clustering approach was used to create “clustergrams”, dendrograms comprising chemical fingerprints of the hair samples at each different temperature. Each clustergram grouped the chemicals in the samples by similarity, allowing the determination of potential sources of exposure. Multivariate factor analysis revealed the presence of phthalates and their corresponding metabolites, confirming that this method can detect biomarkers associated with pollution exposure. This method enables the rapid and sensitive detection of a wide spectrum of toxicologically relevant compounds in human hair, providing an initial screening tool for measuring human exposure and assessing health risks.

## Graphical abstract

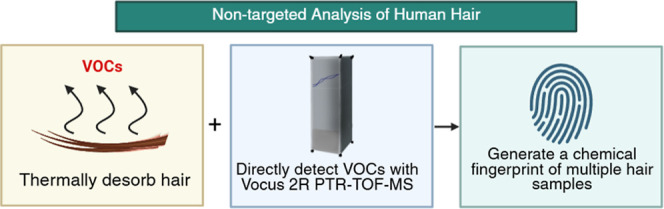

## 1 Introduction

Hair is a unique biological tissue that accumulates trace elements, hormones, drugs, and various organic and organometallic compounds.^1,2^ Through the interaction of the circulatory system and the hair follicle, trace elements present in an organism’s bloodstream are incorporated into the physical structure of the hair at the concentrations they have at the time of hair synthesis.^3,4^ Drawing from the average growth rate of 1 cm per month, hair samples can be sectioned off to determine chemicals present within the body during certain time periods.^5^ Thus, hair samples provide valuable insight into a subject’s chemical exposure from a week to several months depending on the length of the hair.^6^ Hair provides a less invasive and more convenient alternative to traditionally used blood and urine samples, which have comparatively shorter windows of detection and require storage at low temperatures.^7,8^ Existing literature has applied hair analysis to a wide range of airborne pollutants, including both indoor and outdoor exposure to metals, organometallic compounds, and organohalogens.^9,10^

However, this useful form of biomonitoring is considerably limited by current analytical approaches. Hair analysis is typically performed by pulverizing or milling the hair, chemical extraction, and detection using GC–MS or LC–MS.^11,12^ Liquid extraction is a targeted approach, as solvents are selected based on their ability to efficiently extract the desired classes of compounds (e.g., polar vs nonpolar).^13^ Preprocessing the hair is time-consuming, and compounds can be altered during the extraction process, compromising the accuracy and completeness of the analysis.^14^ In addition, compounds present in small concentrations, such as those relevant to evaluating chemical exposure, may not be fully recovered during extraction.

This study proposes a novel method of untargeted analysis to characterize the semivolatile organic compounds (SVOCs) in a hair sample, which are defined as organic compounds with a broad range of vapor pressures between 10^–14^ and 10^–4^ atm.^15^ Using thermal desorption, chemicals in hair can be directly detected without complex pretreatment. This thermal extraction technique is directly coupled with Vocus 2R proton transfer reaction time-of-flight mass spectrometry (Vocus-PTR-TOF-MS) to create a real-time, high-resolution chemical fingerprint of hair samples. Thermal extraction has been previously employed to determine the chemical composition of solid samples, including asphalt binders and contaminated cork wine stoppers.^16,17^ New developments in PTR-MS technology refined over the last several years offer increased sensitivity and the ability to detect even low-volatility VOCs from heated solid samples.^18^ The two prior studies that employ thermal desorption to analyze the chemical content of hair targeted 8 or fewer compounds.^19,20^ This exploratory method is intended as a rapid screening tool to perform nontargeted analysis of a broad range of analytes in human hair, as the Vocus can detect from hundreds to several thousands of molecules in real time.^16,21^ Though this method does not yet allow for absolute quantification of mass fractions in hair, it provides a broad-spectrum analytical approach that could direct future validation with traditional methods, including GC–MS. Here, we report the method which has valuable applications in future studies involving the human exposome, biomonitoring, and air quality.

## 2 Materials and Methods

### 2.1 Hair Sample Preparation

Five hair samples from deidentified donors were used in this study. The specimens were obtained from the vertex region of the scalp. Each bundle of hair strands consisted of approximately 50–90 strands weighing a total of 20 mg. The samples were secured with a piece of cotton twine and then wrapped in aluminum foil and placed in plastic sandwich bags. Although aluminum foil may contain trace amounts of polyfluorinated compounds, our study does not analyze PFAS. Thus, aluminum foil was used in accordance with the Society of Hair Testing guidelines.^22^ Blank samples displayed minimal emissions, and sample concentrations exceeded the aluminum foil background emissions by several orders of magnitude. To prevent microbial growth and minimize volatilization of compounds, the samples were stored in a 4 °C refrigerator.

Before analysis, each hair sample was weighed using a Fisher Science Education Analytical Balance with a draftshield, providing a precision of 0.1 mg. The hair samples were approximately 20 mg each. As the TD-Vocus method is highly sensitive, this mass is considerably less than is required by traditional methods, which typically require 100 mg or more of hair.^23,24^ Samples were placed in small polystyrene Petri dishes. Each sample was assigned a number. Hair Sample 5 was divided into two parts, 5A and 5B, to serve as a comparison between rinsed and unrinsed hair samples. 1000 μL of methanol (≥99.9%, HPLC grade, Sigma-Aldrich) were dispensed onto all samples except 5B using a micropipette to rinse external residue. Excess methanol from each sample was discarded into a waste bottle. Samples were placed in clean Petri dishes and left to dry in a fume hood for 24 h to minimize VOC contamination. Methanol was selected as it removed the majority of externally deposited target compounds without affecting biologically incorporated chemicals.^25,26^

### 2.2 Instrument Analysis

A Vocus 2R proton transfer reaction time-of-flight mass spectrometer (Vocus- PTR-TOF-MS or Vocus) (Aerodyne Ltd., Boston, MA) was used to measure real-time chemical composition of air purged over heated hair which generated steady-state chemical fingerprints of each hair sample at each temperature. The Vocus was configured by coupling with a Gerstel TC2 Tube Thermal Extractor (Gerstel, Germany) directly on the front-end of the Vocus and combining with a Gerstel C200 controller for an automated 3-step thermal desorption system. The Vocus recorded emissions measurements within the mass-to-charge ratio (*m*/*z*) range of 300,000–100,000 (Th) at a time resolution of 1 s, subsequently averaged to 5 s. The temperature was 120 °C, the pressure was 2.3 mbar, the voltage was 596 V, and the drift tube was 10 cm. The *E*/*N* ratio, which characterizes the strength of the drift field (*E*) and density (*N*), was maintained constantly at 141 Td.^27^ This is at the high end of the *E*/*N* range, which prevented clustering of compounds with water molecules. A nitrogen (N_2_) gas cylinder was connected to a mass flow controller (MFC) using polyetheretherketone (PEEK) tubing with an outer diameter of 1/16 in. keeping a constant flow rate of 400 standard cubic centimeters per minute (sccm). The MFC was connected to the Gerstel TC2 Tube Conditioner using 1/16 in. PEEK tubing. The Gerstel was attached directly to the Vocus, which subsampled ∼200 sccm of the outgoing gas flow. Excess flow (flow greater than 200 sccm) was overflowing to an LI-850 CO_2_/H_2_O gas analyzer.

Prior to the VOC measurements, glass tubes were washed thoroughly in a laboratory glassware washer using a fragrance-free detergent, rinsed with deionized water, and baked in an oven overnight for at least 8 h at 200 °C. For hair samples, the programmed sequence was as follows: ramp to 60 °C, hold for 5 min at 60 °C, ramp to 90 °C, hold for 5 min at 90 °C, ramp to 120 °C and hold for 10 min. Blank control measurements were conducted before processing the hair samples, with only a blank tube inserted and thermally desorbed to assess background concentrations. The tube was inserted into the Gerstel TC2 and the temperature ramp began. This process was repeated consistently for each hair sample.

### 2.3 Quality Assurance and Quality Control

Both the air in the laboratory and the glass tube in which samples were held have potential to influence the chemical fingerprint of each sample with trace-level impurities. To mitigate this, a clean and empty glass tube was inserted into the Gerstel TC2 Tube Conditioner and was processed using the same thermal ramp as the hair samples. To assess the degree to which the superficially adsorbed compounds contribute to emissions from hair, each sample was rinsed with methanol, reserving half of a single hair sample as an unrinsed control. As the flushing carrier N_2_ gas does not contain CO_2_, the CO_2_ signal measured by LI-850 aided in confirming the lack of leaks from air in the laboratory and complemented measurement of water vapor emission from hair.

### 2.4 Data Analysis

Following the thermal desorption of the hair samples, the resulting data were processed in the Interactive Data Language (IDL) using a PTRwid software comprising a set of processing routines capable of autonomous and accurate mass scale calibration as well as the computation of a “unified mass list”.^28^ This list is compiled by baseline signal computation and peak shape analysis to set boundaries and to correct overlapping peaks. Through this process, *m*/*z* ratios are accurate within 1 mDa which aids the assignments of chemical formulas.

As with any mass spectrometric technique, the molecular formulas assigned in this study may represent different isomers. In this study, we refer to likely identities based on contextual consistency and number of literature references in the ChemSpider database. This method is best considered as a nontargeted screening tool, and we recommend complementary verification of dominant isomers using offline techniques such as GC–MS (with derivatization) or LC–MS. The major advantage of our method is real-time assessment of the signal and immediate fingerprinting of chemical formulas. In addition to the *m*/*z* ratios identified automatically from the PTRwid’s output and subsequently verified, it was assumed that the detected mass represents the monoisotopic mass in addition to a proton which was used to assign a chemical formula manually using ChemSpider.^29^ MATLAB version R2023a was used alongside the bioinformatics toolbox to assess the real-time signals, the abundance filter of 1 ppt to retain 1392 compounds with sufficiently high signal-to-noise ratio. The matrix of fingerprints was fed to a hierarchical clustering algorithm which groups compounds across the samples by chemical similarity, providing valuable insights for sample similarity and chemical source identification.^16,30^ These were generated for the steady-state emission periods from hair samples thermally extracted at each 60 °C, 90 °C, and 120 °C temperature level. The resulting graphs, called “clustergrams”, contain the top 100 compounds within the hair data set.

## 3 Results and Discussion

### 3.1 Method Validation

A standard of deuterated phthalate was used as an internal calibrant to validate the proposed method for measuring small concentrations of VOCs in hair. Deuterated phthalate was selected because of the relevance of phthalates in human exposure studies. Initially, the standard was added to each hair sample to gauge the method’s sensitivity, but sufficient evaporation was not achieved during the time frame of the thermal desorption. To back calculate sensitivity, a 0.1 μL standard of deuterated dibutyl phthalate with a density of 1.158 g/mL at 25 °C (Sigma-Aldrich) was evaporated at 120 °C for 16 h in a glass tube, yielding an 86.6% recovery. Deuterated dibutyl phthalate was selected because it is not naturally found in hair, but is chemically similar to other phthalates, which are frequently the focus of hair studies related to human health.^31,32^ Additional details are provided in Section 1 of the Supporting Information.

To assess reproducibility, a bulk hair sample (designated Sample A) was divided into five subsamples (A1-A5). To evaluate interday reproducibility, Sample A1 was analyzed on Day 1 (*t* = 0 h.), Sample A2 was analyzed on Day 2 (*t* = 15 h.), and A3 was analyzed on Day 3 (*t* = 45.5 h.). Intraday reproducibility was assessed on Day 3 by analyzing samples A3 and A4 (*t* = 56 h.).

Each replicate was thermally desorbed for 25 min at 90 °C. To demonstrate reproducibility across different *m*/*z* ratios, three VOCs were chosen: C_5_H_6_O, C_4_H_7_ON, and C_9_H_14_O. These compounds were selected for their relevance to human exposure and were tentatively identified as methylfuran, methacrylamide, and isophorone. Methylfuran exposure can occur from vehicle exhaust and cooking, methacrylamide exposure can arise from industrial emissions, and isophorone is common within building materials, plasticizers, and paints. As exhibited in Figure 1, the similarity of the thermal-extraction signal profiles over time demonstrates the interday and intraday reproducibility of the method, closely following the real-time signals and quadruple replication, with expected minor temporal variation between subsamples.

**Figure 1:**
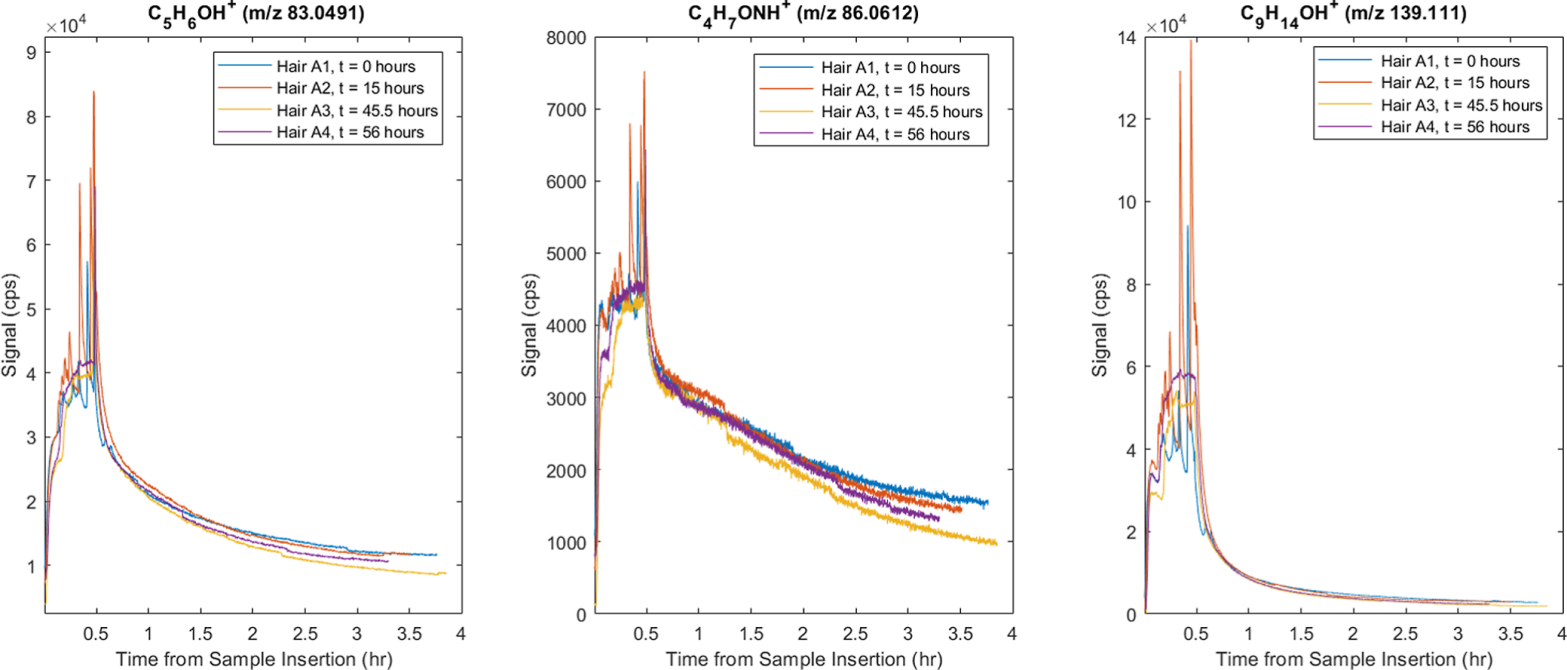
Average signal intensity (in cps) is shown across all subsamples of Hair A for three ions, tentatively identified as C_5_H_6_O (methylfuran), C_4_H_7_ON (methacrylamide), and C_9_H_14_O (isophorone). The consistency of signal across several days supports both interday and intraday reproducibility of the method.

As further complemented in Bland–Altman plots (Figure S1), the relative standard deviation (RSD) values were 11.08% for methylfuran, 9.71% for methacrylamide, and 15.20% for isophorone, confirming close reproducibility of a complex natural matrix. Though no previous study has applied Vocus PTR-TOF-MS to human hair, Zhang et al. (2022) utilized TD-ESI/MS to rapidly desorb and analyze β-agonists in animal hair, yielding a similar range of 7.2–14.6%, reinforcing that our RSDs are near the general range for thermal-desorption based hair analysis.^19^

It is also crucial to discern between compounds emitted from the hair itself and superficially adsorbed compound emissions in order to measure trace level contaminants. These potential sources of interference include air inside of the laboratory getting into contact with the sample during insertion of the glass tube, potential trace emissions from the glass tubes holding the sample during heating, and external residues from hair care products and the environment.^33^ The methanol rinse applied to all samples except for 5A removed compounds associated with common hair residues, such as laureth-2 (C_16_H_34_O_3_), a surfactant used in shampoo, and oleic acid (C_18_H_34_O_2_), a fatty acid found in human sebum (Supporting Information Figure 3).

Although the glass tubes were washed and heated to prevent contamination, their blank trace-level emissions were recorded as a quality control measure. To do so, three empty glass tubes were heated from 60 to 120 °C using the same temperature ramp as the hair samples. Emissions from the background and the blanks were much lower than those from the samples and can be considered negligible (Supporting Information Figure 1). This suggests that most emissions that have been discussed should be attributed to hair.

### 3.2 Emission Profiles of Phthalates During Thermal Desorption

The analytical method developed here to assess the chemical content of hair revealed a myriad of compounds which may be relevant to human health, including xenobiotic compounds and endogenous biomarkers. The experiment was focused on an untargeted analysis that did not preselect, but detected the broad mass range comprising thousands of compounds. Comprehensive factor analysis of the emissions from hair revealed 8 distinct factors, each containing compounds of relevance to human health (Supporting Information Table 1). For example, Factor 1 contained several phthalates, which are endocrine disruptors frequently found in the environment as plasticizers.^34^ Prior studies have used human hair analysis to examine phthalate exposure, typically relying on liquid chromatography-tandem mass spectrometry (LC–MS).^35^ Figure 2 displays the normalized emission rates of several selected phthalates in a single hair sample over time at different temperature hold values.

**Figure 2:**
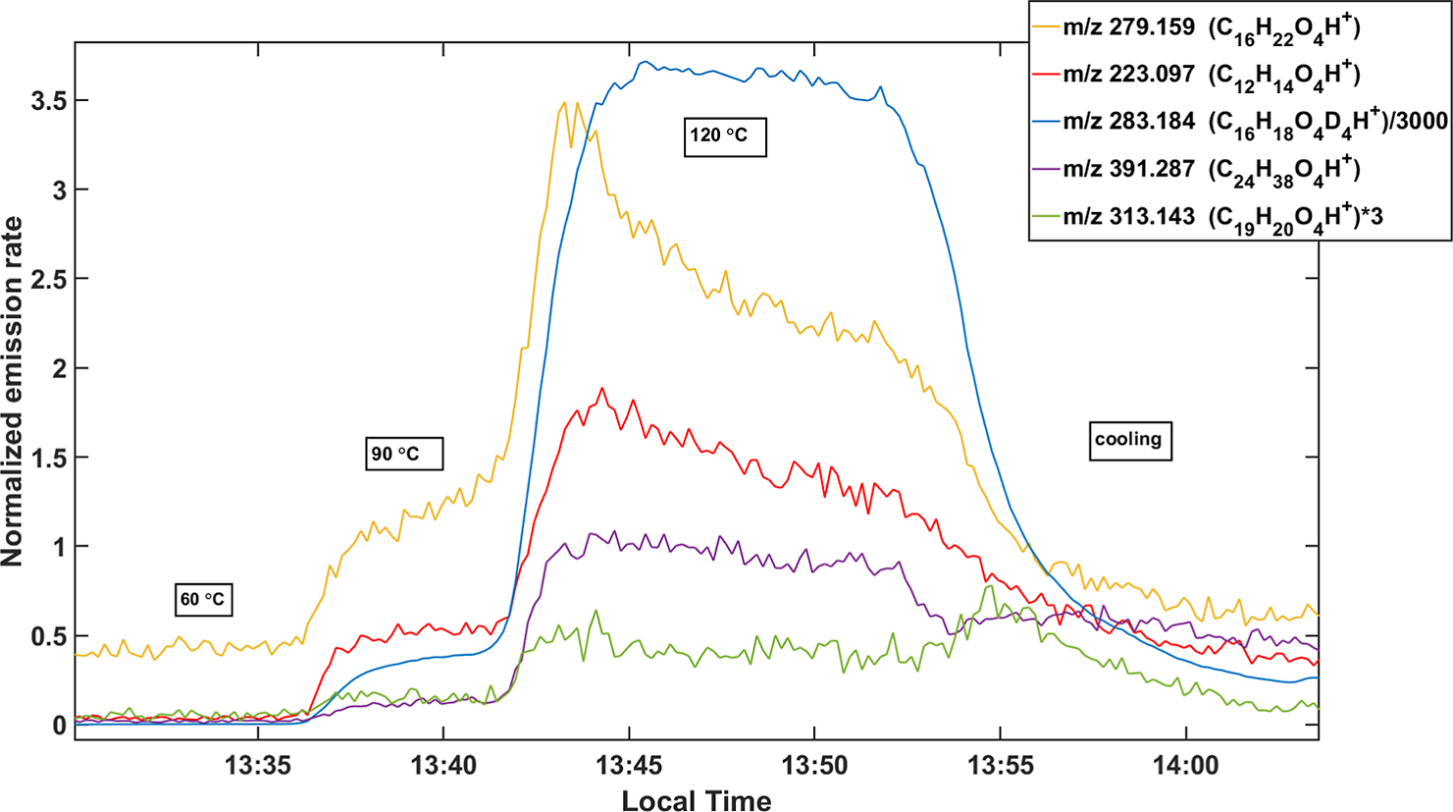
Behavior of selected phthalates during thermal desorption.

The selected compounds include dibutyl phthalate (C_16_H_22_O_4_), diethyl phthalate (C_12_H_14_O_4_), diethylhexyl phthalate (C_24_H_38_O_4_), benzyl butyl phthalate (C_19_H_20_O_4_), and deuterated dibutyl phthalate (C_16_H_18_O_4_D_4_), which was a standard added to ensure instrument sensitivity. In addition to the phthalates in Figure 2, the hair samples also contained monobutyl phthalate (MBP), the metabolite of dibutyl phthalate (DBP) and a secondary metabolite of benzyl butyl phthalate (BBP).^36^ The detection of metabolites helps distinguish between endogenous and exogenous contamination in hair, which is an obstacle when using hair to measure exposure to organic compounds.^37^

There is a clear connection between temperature and normalized emission rate, highlighting the integral role of thermal desorption in this method. Emission rates remain relatively stable at 60 °C, but increase steadily over time at 90 °C as phthalates are liberated from the matrix of the hair sample. However, once the samples were heated to 120 °C, emission rates generally decreased as the phthalates were depleted from the hair matrix. The relation between desorption temperature, emission rate, and *m*/*z* ratio is further detailed in Figure S5 of the Supporting Information.

The compounds detected span multiple orders of magnitude in volatility. As temperature increases, the fraction of the compound in the gas phase increases relative to the condensed phase.^38^ Thus, thermal desorption plays a central role in the detection and analysis of SVOCs in solid samples. Furthermore, careful optimization of desorption temperature is necessary to detect compounds of interest without causing chemical decomposition or denaturing the hair sample.

### 3.3 Chemical Fingerprints

Emission measurements obtained during thermal desorption were used to generate chemical fingerprints. The fingerprints for three of the hair samples at 90 °C are depicted in Figure 3, which demonstrates the method’s usefulness during untargeted and complex chemical analyses. Figure 3 displays the chemical makeup of samples HAIR1, HAIR2, and HAIR3 at a constant temperature of 90 °C, including hundreds of compounds across a broad range of volatilities. Instrument calibrations were performed to derive a fit between sensitivity and proton transfer reaction (*k*_PTR_) coefficient to estimate compound-specific sensitivities by which the measured signals (cps) were divided to calculate concentrations (ppb)^18^ of the *m*/*z* spectrum. Further details on calibration are provided in Table S1.

**Figure 3:**
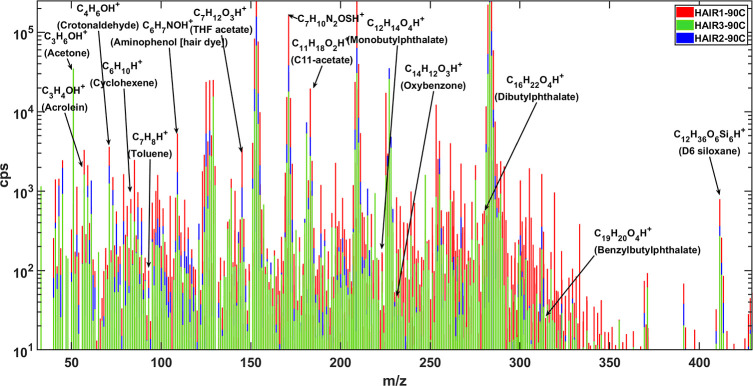
Chemical fingerprints of HAIR1, HAIR2, and HAIR3 at 90 °C.

The full mass spectrum represents untargeted analysis of observed emissions. The data mining process may be focused on specific compounds of interest (e.g., in forensic applications), or alternatively, those which may be most abundant, most toxic, or specific to certain sources. Several compounds labeled in Figure 3 can be relevant to human health. These include the previously mentioned phthalates: benzyl butyl phthalate, dibutyl phthalate, and monobutyl phthalate. Several compounds consistent with a personal care product source included D6 siloxane, oxybenzone, and aminophenol. The unique chemical profile of each sample gives insight into each individual’s chemical exposure, bolstering the usefulness of the proposed method in exposome studies.

### 3.4 Clustergrams for Sample Comparison and Source Attribution

Chemical fingerprints were further analyzed using clustergrams, which showcased the complex emission profiles of the hair samples. Hierarchical clustering is used to group compounds present within the hair based on similarity, which aids in attributing chemicals to a potential source of exposure. The resulting graphs, called clustergrams, depict the extent to which compounds are expressed in a sample on a heatmap, and a dendrogram reflects the level of similarities at different levels. Figure 4 contains the clustergram for the hair samples at 90 °C performed on 100 (out of 1392) compounds. Each column of the heatmap corresponds to one chemical compound in the data set while each row represents an individual sample.

**Figure 4:**
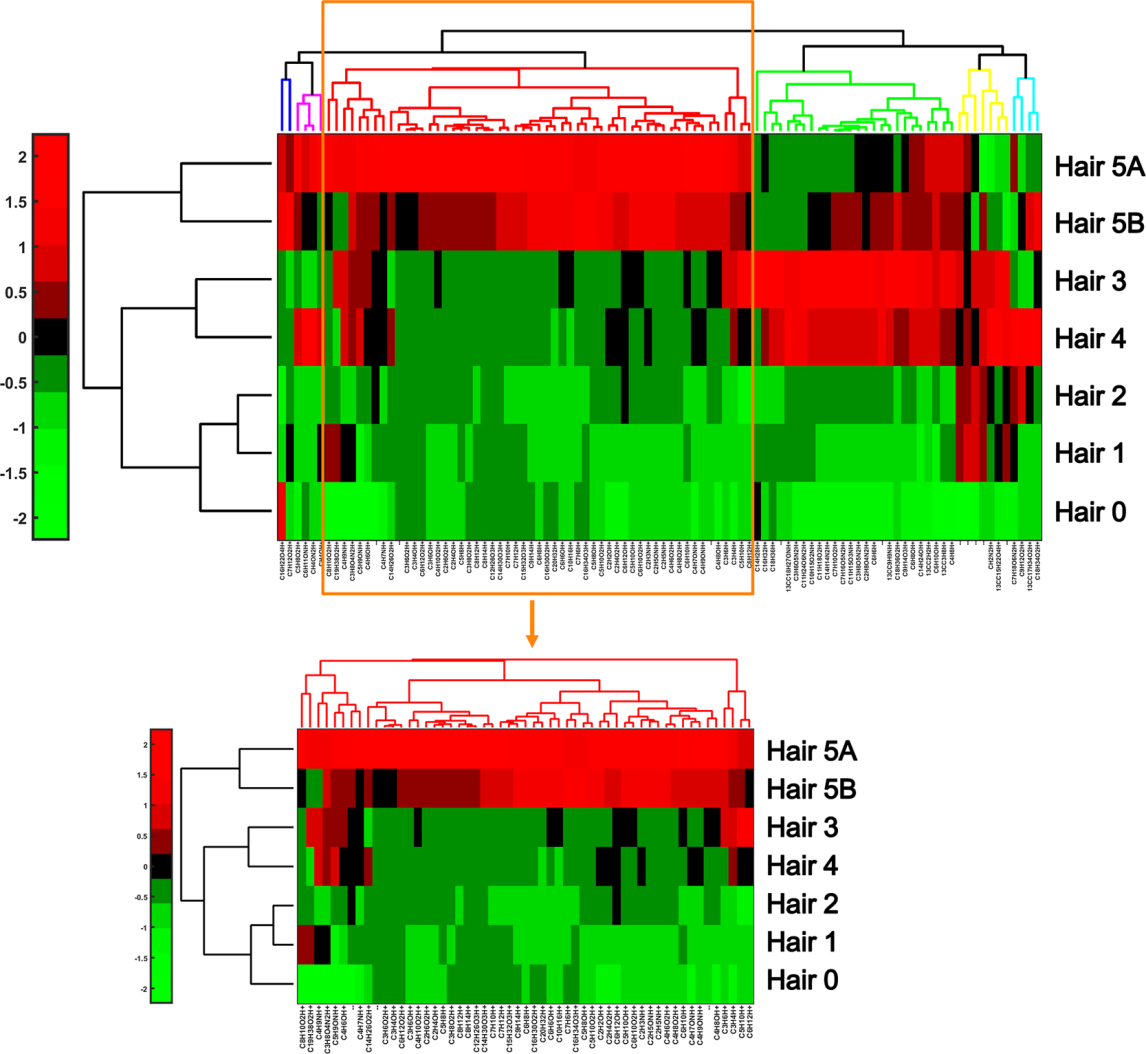
Clustergram of compounds released by hair samples at 90 °C and a subsection of the dendrogram containing compounds consistent with air pollution. Formulas and tentative compound identities are indicated in Table S3.

The heatmap reflects the magnitude of expression of compounds using green, black, or red coloration. Each row of the heatmap corresponds to a hair sample. Green corresponds to a level of chemical expression below the mean of the data set, implying that the sample exhibits a lower concentration of the compound relative to most other samples. Black indicates an average level of expression of a compound. Red indicates a level of chemical expression higher than the mean of the data set.^39^ Similar samples will typically exhibit similar coloration within their rows. Thus, these diagrams also facilitate visual comparison between the unique chemical profiles of each sample, allowing viewers to pinpoint possible differences. The clustergram in Figure 3 displays the degree of chemical expression within each hair sample, suggesting a high level of chemical complexity. Each row of the clustergram differs significantly, as each subject from whom the hair was gathered had a unique array of chemical exposures. When paired with corresponding demographic data, these clustergrams could be used to identify patterns and risk factors in exposure studies.

Clustergrams can also aid in the source attribution component of exposure studies. The colorful branches on the top of the clustergrams, known as dendrograms, group the compounds in the data set by chemical similarity. Subsections of the dendrogram may be used to identify a potential source of a subject’s chemical exposure, which could be confirmed by demographic data or air quality measurements near the subject’s residence. Figure 4 isolates a particularly interesting portion of the clustergram, which displays chemical formulas consistent with exposure to air pollution. Several of these compounds can be associated with cigarette smoke, such as crotonaldehyde (C_4_H_6_O), acetaldehyde (C_2_H_4_O), and acrolein (C_3_H_4_O).^40^ Additionally, we identified a formula consistent with cotinine (C_10_H_12_N_2_O), a major metabolite of nicotine, which is graphed in Figure S6. These compounds are consistent with possible exposure to tobacco smoke, which could occur due to actively smoking or through passive second-hand exposure. However, no causal attribution can be made, as smoker status was not disclosed in this pilot study. This dendrogram also includes cyclohexane (C_6_H_12_), which has been found in the breast milk of women living in polluted areas.^41^

### 3.5 Limitations and Future Applications

The proposed method presents a complementary alternative to traditional liquid extraction and GC–MS, facilitating the rapid and nontargeted detection of an extensive range of VOCs using thermal desorption and the Vocus 2R PTR-TOF-MS. This exploratory approach allows for time-efficient, comprehensive screening of solid samples without solvent-based liquid extraction, with many promising applications within the fields of air quality, human health, and forensics.

Though this method provides a strong foundation for further analytical work, several limitations should be addressed. As the method is designed for rapid screening, it is not yet intended for absolute quantification of all VOCs in hair without reference standards. However, the method could be used to identify compounds of interest to exposure studies and undergo subsequent validation using more established methods like liquid extraction and LC–MS or GC–MS. Future studies should also determine the extent to which pigment variability affects the chemical content of hair, as pigmented hair may display higher compound binding ability compared to nonpigmented hair.^42^ This may include controlled animal studies to establish the relationship between chemical exposure to chemical levels in hair of various pigmentation. Further analysis should be conducted to measure and optimize the removal of surface contamination prior to desorption. Similarly, the impacts of grinding or milling hair compared to the thermal desorption of intact hair samples should also be evaluated.

With further refinement, this method shows promise in the context of high-volume solid sample analysis related to human exposure. It could be used alongside pollution monitoring bracelets to estimate exposure that occurs indoors, as measurement of indoor exposure to pollutants is a burgeoning area of interest within the field of air quality. In future works, reference ranges could be determined for certain compounds, providing a link between exposure profiles and health outcomes.

## Supplementary Material



## Notes

Bland–Altman plots for methylfuran, methacrylamide, and isophorone. Description of phthalate response calibration and graph of deuterated phthalate evaporation. Dendrogram at 60 °C of a group of compounds possibly associated with common hair residues, including shampoo and sebum (PDF)

## †

Johns Hopkins University, Baltimore, MD 21218, USA
