# Supplementary material for: Ultrasensitive Direct Chemical Analysis of Human Hair Using Proton Transfer Reaction Time-of-Flight Mass Spectrometry (PTR-TOF-MS) for Nontargeted Exposure Profiling
Source: Chem Res Toxicol. 2025 Sep 8;38(10):1681–8. doi: 10.1021/acs.chemrestox.5c00002 (PMC12541802; doi:10.1021/acs.chemrestox.5c00002)
Supplement: Supplementary file 1 [file tx5c00002_si_001.pdf]

SUPPORTING INFORMATION

Ultrasensitive direct chemical analysis of human hair using Proton Transfer Reaction Time-of-Flight Mass Spectrometry (PTR-TOF-MS) for non-targeted exposure profiling

Anna C. Neville[1*], *David A. Jarma*[1], *Daniel C. Blomdahl*[1,2], *Chou-Hsien Lin*[1], *Kerry A. Kinney*[1], *and Pawel K. Misztal*[1*]

[1]Maseeh Department of Civil, Architectural and Environmental Engineering, University of Texas at Austin, Austin, TX 78712, USA

[2]Now at: Department of Environmental Health and Engineering, Johns Hopkins University, Baltimore, MD 21218, USA

*Corresponding authors: Anna Neville (acnevi@utexas.edu); Pawel K Misztal (misztal@utexas.edu)

Summary: 9 pages, 6 figures, 1 table

Table of Contents

Section S1. Bland-Altman Plots

Figure S1. Bland-Altman plots for methylfuran, methacrylamide, and isophorone

Figure S2. Emissions from blank tubes

Section S3. Calibrating phthalate response

Table S1. List and properties of compounds contained in the calibration mixture.

Figure S3. Emission rate of deuterated phthalate over 16 hours at 120 °C

Table S1. List and properties of compounds contained in the calibration mixture.

Figure S4. Dendrogram at 60 °C of a group of compounds possibly associated with common hair residues, including shampoo and sebum.

Figure S5. Thermogram of a hair sample during thermal desorption from 60-90 °C

Figure S6. Chemical signal of $C_{10}H_{12}N_2OH+$, a formula consistent with cotinine. Cotinine is commonly associated with tobacco smoking.

Table S2. Factor analysis of selected compounds

Table S3. Compound formulas and tentative identification for the highlighted subsection of the graph of compounds consistent with air pollution.

Section S1. Bland-Altman Plots

Figure S1 depicts Bland-Altman plots of the normalized signal intensities for all subsamples. These plots display the degree of agreement of each measurement relative to the mean of the entire group. The relative standard deviation (RSD) values were 11.08% for methylfuran, 9.71% for methacrylamide, and 15.20% for isophorone, confirming acceptable inter and intra-reproducibility.

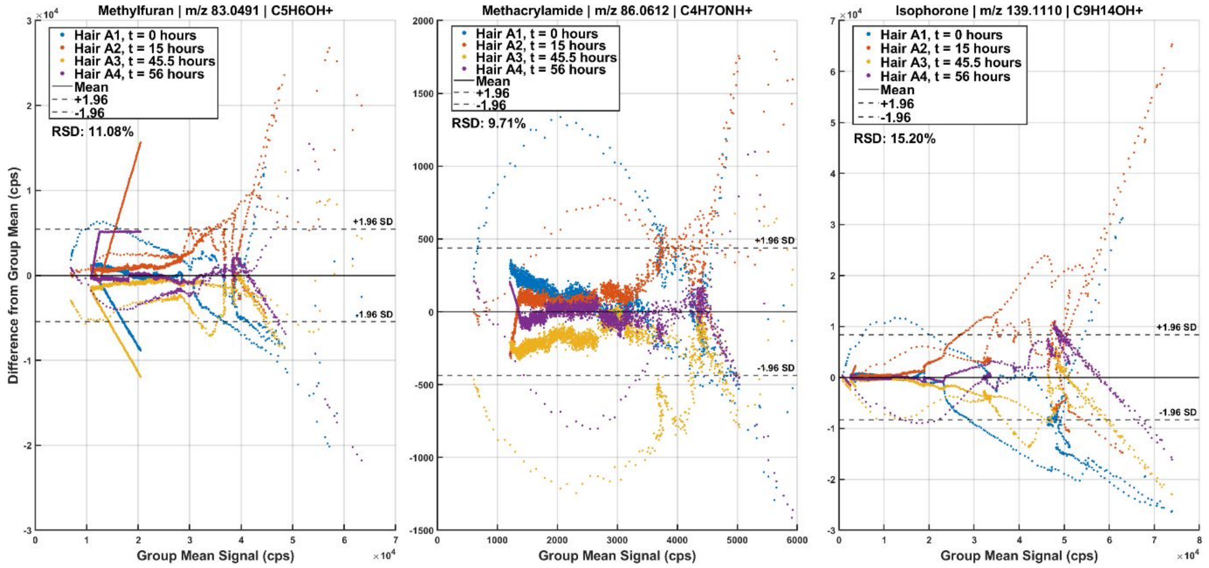

Figure S1. Bland-Altman plots of 2-methylfuran (C_5H_6O), methacrylamide (C_4H_7ON), and isophorone ($C_9H_{14}O$) displaying the reproducibility of VOC signal intensity (cps) measurements across four hair sample replicates undergoing thermal desorption. The x-axis denotes the group mean signal intensity (cps) for the set of replicates, while the y-axis shows the difference between each individual replicate and the group mean signal (cps). The solid line in the center denotes the

group mean signal (cps), while the two dashed lines indicate standard deviations of +1.96 and -1.96.

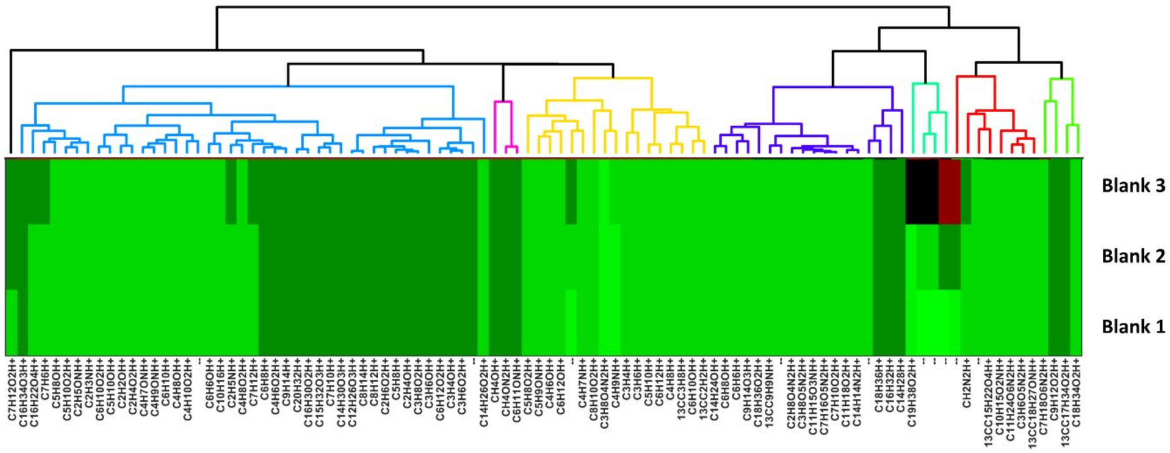

Figure S2. Background emissions associated with the empty glass tubes at 90 °C.

Section S3. Compound Calibration

In terms of explicit calibration of phthalate response, 0.1 μL of dibutyl phthalate standard was added to a clean glass tube, which was heated in the thermal desorption unit for 16 hours and measured using the Vocus 2R PTR-TOF-MS. The emission rate of deuterated phthalate (g/s), shown in Figure 1, was integrated with respect to time to determine the total mass thermally extracted over 16 hours. This method yielded a measured amount of 91.63 μg of deuterated phthalate, compared to the actual mass of 105.8 μg. This translates to an 86.6% recovery rate for deuterated phthalate, which is within the bounds of reported uncertainty and can be attributed to the standard residue not fully evaporated during the experiment's desorption time and

temperature. Increasing desorption temperature could further increase this recovery, but prior literature suggests a maximum of 230 °C to prevent thermal decomposition of the hair[1]. Deuterated dibutyl phthalate has a boiling point of 340 °C, but can be detected at a lower temperature, as thermal desorption of sVOCs and VOCs does not require a compound to reach its boiling point. Rather, thermal desorption increases vapor pressure using elevated temperature and a steady flow of carrier gas.

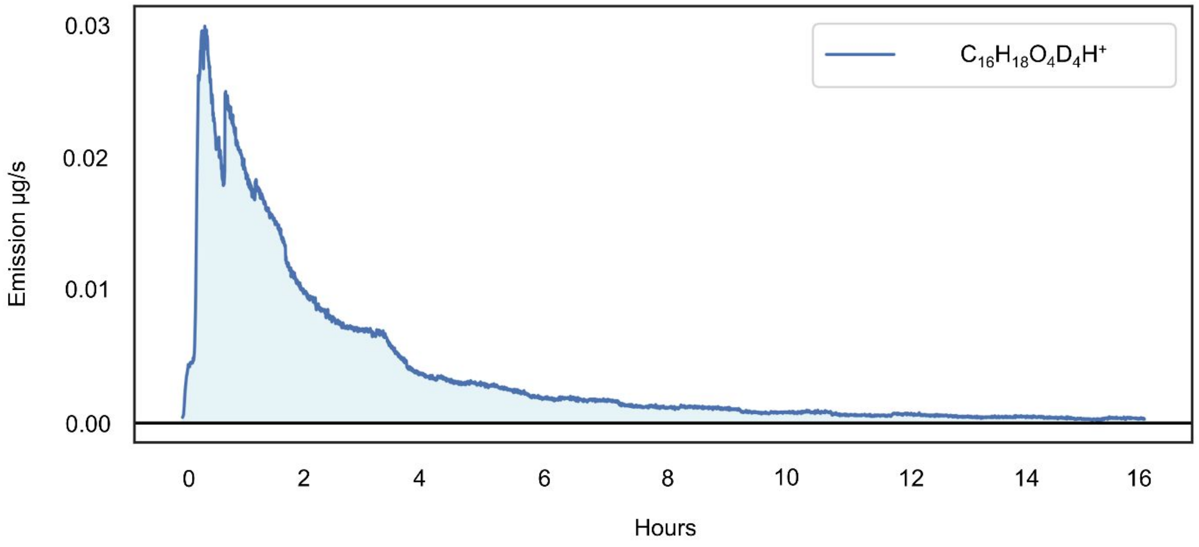

Figure S3. Emission rate of deuterated phthalate over 16 hours at 120 ˚C.

Average abundance (ppb) values were calculated using calibration with authentic gas standard (Apel-Riemer Environmental) containing a mixture of different calibrants spanning molecular weights from 33 to 450 amu. The contents of the calibration mixture can be found below in Table S1.

Table S1. List and properties of compounds contained in the calibration mixture.

Compound	CAS#	Concentration (ppb)	Boiling Point (˚C)
Methanol	67-56-1	541	63.9
Acetonitrile	75-05-8	491	82.1
Propanal	123-38-6	477	47.9
Benzene	71-43-2	510	80.1
Hexanal	66-25-1	499	131.0
Hexamethylcyclotrisiloxane (D3)	541-05-9	501	134.0
m-Xylene	108-38-3	492	139.1
Styrene	100-42-5	499	145.2
α-Pinene	80-56-8	127	155.0
β-Pinene	18172-67-3	121	165.0
Octamethylcyclotetrasiloxane (D4)	556-67-2	502	175.0
3-Carene	13466-78-9	118	170.0
p-Isopropyltoluene	99-87-6	470	176.0
Limonene	5889-54-8	130	176.0
Acetophenone	98-86-2	482	202.0
Decamethylcyclopentasiloxane (D5)	541-02-6	502	210.0
Dodecamethylcyclohexasiloxane (D6)	540-97-6	181	245.0

These explicit calibrations are subsequently used to create a plot correlating calibrant sensitivities (cps/ppbv) and their proton-transfer reaction rate coefficients (kPTR). To estimate concentration of the broad mass spectrum, these plots were used to estimate the analyte concentrations for species not included in the calibration gas[2,3]. This method results typically in 30% uncertainty. The internal standard was used in addition to complement understanding of how phthalates behave as a function of temperature – however, internal spiking is not required by this direct method as opposed to solvent extraction methods, where recoveries can substantially differ. Further advantage of using gas-phase calibration as opposed to spiking is that spiking cannot represent compounds that are built-in hair during its growth but can serve as a representation of exogenic and surficial deposits.

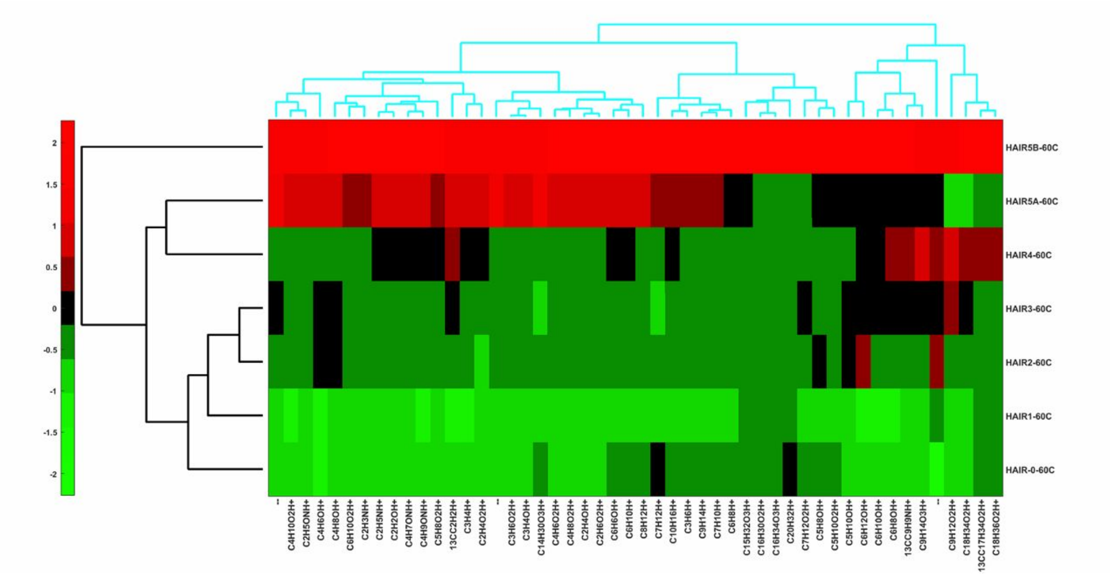

Figure S4. Dendrogram at 60 °C of a group of compounds possibly associated with common hair residues, including shampoo and sebum.

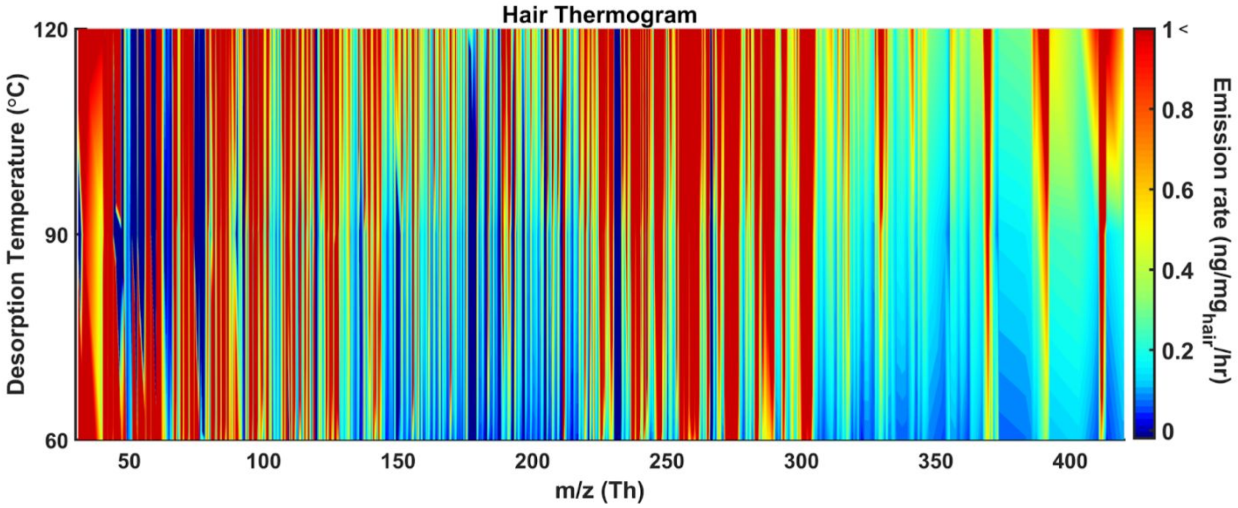

Figure S5. Thermogram of a hair sample between the thermal desorption temperatures of 60-90 °C. In general, high volatility compounds (mostly left part of the *m/z* spectrum) rapidly evaporate early during the desorption process, while some heavier SVOCs tend to increase emission later as a function of temperature.

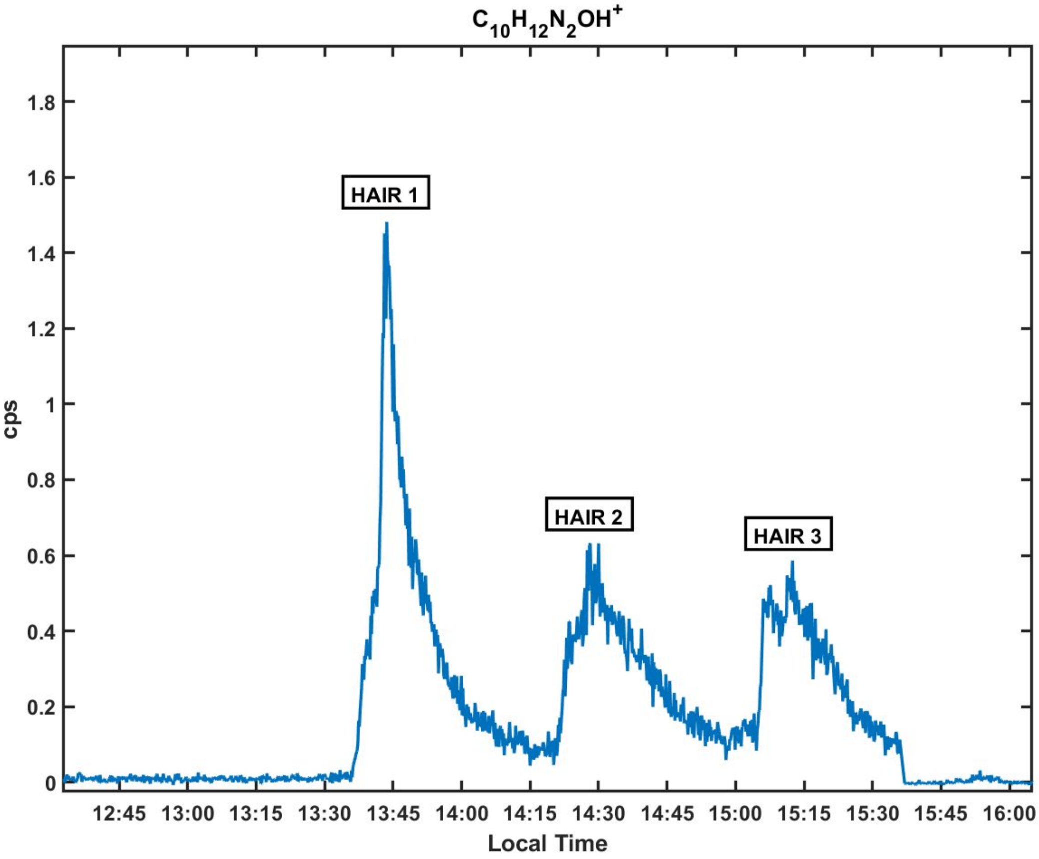

Figure S6. Chemical signal of $C_{10}H_{12}N_2OH+$, a formula consistent with cotinine. Cotinine is commonly associated with tobacco smoking.

Table S2. Factor analysis of selected compounds, focusing on those hazardous to human health and markers of pollution. F1, F2, and F3 denote Factor 1, Factor 2, and Factor 3 in the multi-factor solution. Each factor represents a grouping of compounds that most closely co-vary over the course of the experiment, compared to the rest of the dataset. Hazard designations are as follows: Flammable, Acute Toxic, Irritant, Health Hazard, Environmental Hazard, and Reproductive Hazard.

Mass	Formula	Presumed ID	Hazards	Avg. Abundance (ppb)	F1	F2	F3	F 4	F 5	F 6	F 7	F 8
313.143	C19H20O4H+	Benzyl Butyl Phthalate (plasticizer for PVC)	Health, Env., Reproductive	0.271	0.69	0.54						
279.159	C16H22O4H+	Dibutyl phthalate (plasticizer)	Health, Env.,	3.13	0.69				0.55			
229.086	C14H12O3H+	Oxybenzone (sunscreen)	Irritant, Env.	0.515	0.4		0.86					
223.097	C12H14O4H+	Monobutyl Phthalate	Health	1.75	0.63				0.6			
147.065	C6H10O4H+	Adipic acid (plastics and foams)	Irritant	1.43	0.65	0.44	0.56	0.56				
139.111	C9H14OH+	Isophorone (solvents and pesticides)	Irritant, Health	3.4	0.95							
115.075	C6H10O2H+	2,5-Hexanedione (human metabolite of hexane)	Irritant, Health	1.88	0.68	0.43		0.57				
107.084	C8H10H+	Xylene (industrial solvent)	Flammable, Irritant, Health	1.61	0.92							
106.072	C7H8H+	Toluene (solvents, glue, paint)	Flammable, Irritant, Health	7.68	0.86							
101.06	C5H8O2H+	Acetylacetone (solvents, paint, tobacco)	Flammable, Irritant	3.34	0.65	0.47		0.55				
100.076	C5H9NOH+	1-Methyl-2-pyrrolidinone (batteries, cleaning products)	Irritant, Health, Reproductive	1.84	0.43	0.72		0.52				
95.0492	C6H6OH+	Phenol (nylon manufacturing, disinfectant)	Corrosive, Acute Toxic, Health	2.12	0.86		0.3					
85.1012	C6H12H+	Cyclohexane (nylon manufacturing, cleaning products)	Flammable, Irritant, Health, Env.	3.93	0.51				0.31	0.76		
83.0854	C6H10H+	Cyclohexene (petroleum)	Flammable, Acute Toxic, Irritant, Health, Env.	5.98	0.88							
79.0539	C6H6H+	Benzene (petroleum, gasoline, tobacco smoke)	Flammable, Irritant, Health	4.96		0.94						
72.0448	C3H5NOH+	Acrylamide (solvents, polymers, tobacco smoke, dyes, fried food)	Acute Toxic, Health	0.56		0.38	0.49					
71.0495	C4H6OH+	Crotonaldehyde (tobacco smoke, gasoline)	Flammable, Corrosive, Acute Toxic, Irritant, Health, Env.	8.5		0.64		0.68				
57.0335	C3H4OH+	Acrolein (tobacco smoke, gasoline)	Flammable, Corrosive, Acute Toxic, Env.	40							0.33	
45.0336	C2H4OH+	Acetaldehyde (tobacco smoke, gasoline)	Flammable, Irritant, Health	36.1	0.75	0.34		0.53				

Table S3. Compound formulas and tentative identification for the highlighted subsection of the graph of compounds consistent with air pollution. The table also includes the number of literature references for each compound on the ChemSpider database.

Formula	Compound Name	# Data Sources from ChemSpider
$C_8H_{10}O_2H+$	Tyrosol	91
$C_{19}H_{38}O_2H+$	Nonadecylic acid	75
C_4H_9NH+	Pyrrolidine	73
$C_3H_8O_4N_2H+$	Methylhydrazine oxalate	7
C_5H_9ONH+	Piperidone	81
C_4H_6OH+	Crotonaldehyde	74
C_4H_7NH+	Isopropyl cyanide	61
$C_{14}H_{26}O_2H+$	Myristoleic acid	56
$C_3H_6O_2H+$	Propionic acid	103
C_3H_4OH+	Acrolein	83
$C_6H_{12}O_2H+$	Hexanoic acid	94
C_3H_6OH+	Acetone	89
$C_4H_{10}O_2H+$	Butanediol	71
$C_2H_6O_2H+$	Ethylene glycol	92
C_2H_4OH+	Acetaldehyde	95
C_5H_8H+	Isoprene	65
$C_3H_8O_2H+$	Propanediol	88
$C_8H_{12}H+$	Cyclooctadiene	56
$C_8H_{14}H+$	Cyclooctene	52
$C_{12}H_{26}O_3H+$	Dibutyldiglycol	52
$C_{14}H_{30}O_3H+$	Hydrocitronellal diethyl acetal	18
$C_7H_{10}H+$	Norbornene	50
$C_7H_{12}H+$	Methylcyclohexene	55
$C_{15}H_{32}O_3H+$	Dodecylglycerol	28
$C_9H_{14}H+$	1,2,3,4-Tetramethyl-1,3-cyclopentadiene	52
C_6H_8H+	Cyclohexadiene	57
$C_{16}H_{30}O_2H+$	Palmitoleic acid	79
$C_{20}H_{32}H+$	Diterpenes	26
C_6H_6OH+	Phenol	103
$C_{10}H_{16}H+$	Monoterpenes	84
C_7H_6H+	Norcaratriene	23
$C_{16}H_{34}O_3H+$	Laureth-2	43
C_5H_8OH+	Cyclopentanone	75
$C_5H_{10}O_2H+$	Methylbutanoic acid	93
C_2H_2OH+	Ethenone	34
$C_2H_4O_2H+$	Acetic acid	111

$C_6H_{12}OH+$	Hexanal	89
$C_5H_{10}OH+$	Pentanal	82
$C_6H_{10}O_2H+$	Acetonylacetone	76
C_2H_3NH+	Acetonitrile	80
C_2H_5ONH+	Acetamide	98
C_2H_5NH+	Aziridine	55
$C_4H_6O_2H+$	Biacetyl	83
$C_4H_8O_2H+$	Butyric acid	109
$C_6H_{10}H+$	Cyclohexene	66
C_4H_7ONH+	Pyrrolidone	70
C_4H_9ONH+	Morpholine	80
C_4H_8OH+	Butanone	80
C_3H_6H+	Propene	55
C_3H_4H+	Propyne	45
$C_5H_{10}H+$	Cyclopentane	60
$C_6H_{12}H+$	Cyclohexane	80

Supporting References

(1) Kong, R.; Li, L.; Liu, W.; Xiang, P.; Zhao, J. Rapid Characterization of Drugs in a Single Hair Using Thermal Desorption Ionization Mass Spectrometry. *Anal. Methods* **2022**, *14* (8), 806–812. https://doi.org/10.1039/D1AY01908B.

(2) Jahn, L. G.; Bhattacharyya, N.; Blomdahl, D.; Tang, M.; Abue, P.; Novoselac, A.; Ruiz, L. H.; Misztal, P. K. Influence of Application Method on Disinfectant Byproduct Formation during Indoor Bleach Cleaning: A Case Study on Phenol Chlorination. *ACS EST Air* **2024**, *1* (1), 16–24. https://doi.org/10.1021/acsestair.3c00011.

(3) Coggon, M. M.; Stockwell, C. E.; Claflin, M. S.; Pfannerstill, E. Y.; Xu, L.; Gilman, J. B.; Marcantonio, J.; Cao, C.; Bates, K.; Gkatzelis, G. I.; Lamplugh, A.; Katz, E. F.; Arata, C.; Apel, E. C.; Hornbrook, R. S.; Piel, F.; Majluf, F.; Blake, D. R.; Wisthaler, A.; Canagaratna, M.; Lerner, B. M.; Goldstein, A. H.; Mak, J. E.; Warneke, C. Identifying and Correcting Interferences to PTR-ToF-MS Measurements of Isoprene and Other Urban Volatile Organic Compounds. Atmospheric Meas. Tech. 2024, 17 (2), 801–825. https://doi.org/10.5194/amt-17-801-2024.
